# Adaptation of Geriatric Interprofessional Education from In-Person to Virtual Simulation

**DOI:** 10.1093/geroni/igab046.003

**Published:** 2021-12-17

**Authors:** Cynthia Hovland

**Affiliations:** Cleveland State University, Cleveland, Ohio, United States

## Abstract

We modified an in-person simulation-enhanced interprofessional education model as necessitated by COVID-19 restrictions to a fully virtual education experience. Online prework remained unchanged but adjustments were made related to previously in-person activities. Diverging from the in-person training we held live virtual poster sessions with learner-presenter interaction. In preparation for their role in the team meeting simulation, learners were moved into preassigned profession-specific breakout rooms for a live virtual huddle with facilitators. Next, learners were moved to preassigned interprofessional breakout rooms where they began the simulated team meeting. After initial discussion, a standardized patient joined the breakout room to present the patient/caregiver perspective. The event ends with a virtual reflective debrief focused on interprofessional collaborative competencies.

